# Ontology-Based Architecture for Intelligent Transportation Systems Using a Traffic Sensor Network

**DOI:** 10.3390/s16081287

**Published:** 2016-08-15

**Authors:** Susel Fernandez, Rafik Hadfi, Takayuki Ito, Ivan Marsa-Maestre, Juan R. Velasco

**Affiliations:** 1 Department of Computer Science and Engineering, Nagoya Institute of Technology, Nagoya 466-0054, Japan; rafik@itolab.nitech.ac.jp (R.H.); ito.takayuki@nitech.ac.jp (T.I.); 2 Department of Computing Engineering, University of Alcala, Madrid 28805, Spain; ivan.marsa@uah.es (I.M.-M.); juanramon.velasco@uah.es (J.R.V.)

**Keywords:** intelligent transportation systems, ontology, reasoning, agents, sensor networks

## Abstract

Intelligent transportation systems are a set of technological solutions used to improve the performance and safety of road transportation. A crucial element for the success of these systems is the exchange of information, not only between vehicles, but also among other components in the road infrastructure through different applications. One of the most important information sources in this kind of systems is sensors. Sensors can be within vehicles or as part of the infrastructure, such as bridges, roads or traffic signs. Sensors can provide information related to weather conditions and traffic situation, which is useful to improve the driving process. To facilitate the exchange of information between the different applications that use sensor data, a common framework of knowledge is needed to allow interoperability. In this paper an ontology-driven architecture to improve the driving environment through a traffic sensor network is proposed. The system performs different tasks automatically to increase driver safety and comfort using the information provided by the sensors.

## 1. Introduction

Intelligent Transportation Systems (ITS) are applications and technological systems created with the aim of improving safety and efficiency in road transportation. These systems allow the control, management and monitoring of the different road elements. Intelligent Transportation Systems can play a major role in reducing risks, high accident rates, traffic congestion, carbon emissions, air pollution, while on the other hand increase safety and reliability, travel speeds, traffic flow and satisfied travelers for all modes. Those systems provide solutions for cooperation and reliable platform for transport. Major areas of ITS in metropolitan deployments are Arterial and Freeway Management [1,2], Freight Management [3,4], Transit Management Systems [5,6], Incident and Emergency Management Systems [7,8,9], Regional Multimodal and Traveler Information Systems [10,11]. Some applications of ITS are: Electronic Toll Collection [12,13], Highway Data Collection [14,15], Emergency Vehicle Preemption [16], Traffic Management Systems [17], Transit Signal Priority [18], Vehicle Data Collection [19], etc.

The continuing evolution of ITS has ushered in a new era of interconnected intelligent systems, which certainly has been a quantitative leap in safety of road transportation. These systems enable the exchange of information between different applications, and the subsequent analysis allows improving the safety of drivers and promotes travel and comfort in road travel.

There are some recent vehicular communication projects, focused in the creation of architectures and standards to allow reliable and extended driving support systems for road safety and transportation efficiency. An example of such efforts is a common reference architecture [20], from the European Telecommunications Standards Institute (ETSI) TC ITS [21] and the International Organization for Standards (ISO) for ITS Communications [22].

One of the most important information sources in ITS is sensors. Sensors can be located into vehicles or as part of an infrastructure element, such as bridges, roads or traffic signs. Sensors can provide information related to the weather conditions and the traffic situation, which is useful to improve the driving process. A sensor network is a network of tiny computers (nodes) equipped with sensors, which collaborate on a common task [23]. These nodes have certain sensory and wireless communications capabilities that enable ad hoc networking without any preset physical infrastructure or central administration. Sensor networks have been used in data acquisition and processing for multiple applications in different fields such as industry, medicine, home automation, military environments, environmental detection, etc.

Ontologies provide a common vocabulary in a given domain and allow defining, with different levels of formality, the meaning of terms and the relationships between them [24]. Due to its high degree of expressiveness, the use of ontologies is crucial to ensure greater interoperability among software agents and different applications involved in Intelligent Transportation Systems. Ontologies facilitate the design of exhaustive and rigorous conceptual schemes to allow communication and information exchange between different systems and institutions.

Although there are several studies that apply ontologies to intelligent transportation systems, most of them do not use information from sensors. The ontology can give a semantic interpretation to information collected by the sensors, facilitating safe driving and improving traffic performance.

This paper presents an ontology-based architecture to improve the driving environment through a traffic sensor network. The system performs different tasks in an automated way to increase the driver safety and comfort using sensor information. For example, such parameters include the air conditioner settings, the traffic lights parameters such as the duration time of the lights and the light intensity, taking into account the traffic flow and the weather conditions.

The paper is organized as follows: Section 2 is a review of the state of the art in ontologies for intelligent transportation systems. Section 3 presents the architecture of the proposed multi-agent system. Section 4 provides different cases studies. Finally, we extract some conclusions and outline future work.

## 2. Related Work

Nowadays there are some recent research efforts focused in the improvement of road safety and traffic efficiency. There are works focused on vehicular communications, such as the work in [25]. This paper presents a two-stage low-pass FIR filter with the main goal of mitigating the effects of adjacent channel interference in vehicular communication systems. Another recent work focused in vehicular communication is T-VNets [26]. It describes a trust establishment architecture for VANETs based on ETSI ITS standard messaging services. The solution can offer high security levels while preserving network resources, allowing detection of different types of active attacks. The restriction of this work is that it is limited only to the ETSI ITS standard and needs to be extended to others.

There are also works related to traffic congestion in ITS. The one proposed in [27] is the ECODE protocol. The aim of this protocol is to evaluate the traffic characteristics and to detect the highly congested road segments in downtown area scenarios. They propose three different execution schemes: reactive, proactive and hybrid. The hybrid scheme balances between the performance of the proactive and reactive schemes in terms of the communication overhead and in terms of the level of traffic evaluation accuracy. The hybrid scheme achieves the best results compared to the others.

In [28], the authors present some considerations related to the number of vehicles, the roadmap topology, and its complexity, as well as the influence of these parameters in vehicular communications. Additionally, they propose the use of a new density metric which allows to accurately characterize the different urban scenarios regarding cooperative Intelligent Transportation Systems. A traffic congestion detection system was presented in [29]. The system allows drivers to identify traffic congestion areas and change their routes accordingly, thus reducing the total CO_2_ emissions and decreasing travel time. In general, the main weakness of these works is the lack of a unified management structure of knowledge, which makes them unable to offer semantic information of the traffic situation beyond the data provided by the sensors. This fact, also hampers interoperability between these systems and other applications involved in the ITS.

In the last years, there has been an increasing interest in ontologies for road transportation systems. In [30], an ontology to represent traffic in highways has been developed. Its aim was the construction of a reliable Traffic Information System providing information about roads, traffic, and scenarios related to vehicles in the roads. It also helps the Traffic Information System analyze how critical a specific situation is. For example, an ambulance may need to know about the congestion status of a toll plaza. Requesting this information is critical if the ambulance is moving to the scene of an accident. On the other hand, if a regular vehicle is moving through a road without hurry, then this information requested is not so critical.

In [31] the authors proposed a high-level representation of an automated vehicle, other vehicles and their environment, which can assist drivers in taking “unorthodox” but practical relaxation decisions (for example when a car damaged does not allow the circulation, take the decision to move to another lane crossing a solid line and overtake the stopped car, if the other lane is clear). This high-level representation includes topological knowledge and inference rules, in order to compute the next high-level motion an automated vehicle should take, as assistance to a driver’s decision making. The principal weakness of this approach is the lack of rules representing prior traffic regulations. They just defined a set of traffic regulations violations, which allow classifying the given motion as “legal” or “illegal”.

In [32] the authors propose an ontology-based spatial context model. The work takes a combined approach for modeling context information utilized by pervasive transportation services: the Primary-Context Model facilitates interoperation across independent Intelligent Transportation Systems, whereas the Primary-Context Ontology enables pervasive transportation services to reason about shared context information and to react accordingly. The independently defined, distributed information is correlated based on its primary-context: location, time, identity, and quality of service. The Primary-Context Model and the associated Ontology are evaluated by modelling a car park system for a smart parking space locator service.

The work proposed in [33] is an approach to create a generic situation description for advanced driver assistance systems using logic reasoning on a traffic situation knowledge base. It contains multiple objects of different type such as vehicles and infrastructure elements like roads, lanes, intersections, traffic signs, traffic lights and relations among them. Logic inference is performed to check and extend the situation description and interpret the situation e.g., by reasoning about traffic rules. The capabilities of this ontological situation description approach are shown at the example of complex intersections with several roads, lanes, vehicles and different combinations of traffic signs and traffic lights. As a restriction, in this work, the passing destination road over the intersection has to be known for each vehicle, so modeling different possibilities according to the real situation of the intersection is not possible.

In [34], the authors propose an ontology for traffic management that combines both traffic concepts and general sensor ontology A3ME [35]. The added concepts are specializations of position, distance and acceleration sensor classes, and the different actions that take place in the car motions. The ontology is developed in OWL, using the JESS reasoner with SWLR [36] rules.

In [37] an ontology-based Knowledge Base, which contains maps and traffic regulations, was introduced. It can be aware of speed situations and make decisions at intersections to comply with traffic regulations, but it does not consider important elements such as traffic signals and weather conditions.

The work proposed in [38] is an ontological model to integrate information from many data sources on ITS. The approach consists in creating a local ontology for each data source, and later integrate the various local ontologies into a single general ontology using different matching algorithms.

A general weakness of ontology-based approaches is that a vehicle and its environment are represented in discrete, symbolic terms: things are true or false but there is no way to represent something intermediate, i.e., a notion of uncertainty. Therefore a first solution to representing uncertainty would be the use of Bayesian Networks, defining the rules as probabilistic dependencies among state variables. In this work we choose a second solution, which consists in restricting the view to describe the context only, providing the right ontology for the current context, and making inferences with certainty about it.

## 3. System Architecture

**Figure 1:**
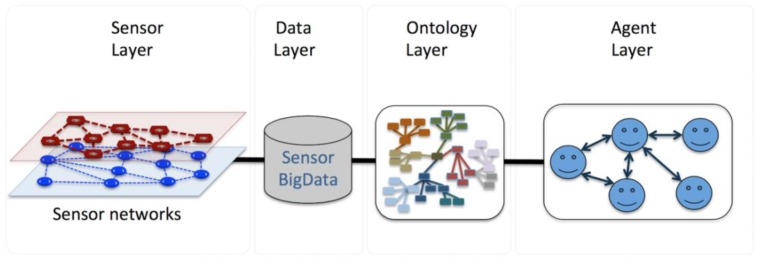
System Architecture.

The proposed system is a four-layer architecture. As shown in Figure 1, the first layer is the sensor network layer, which has been deployed along the road infrastructure. These sensors will sense different measures values, such as traffic flow and weather conditions. The second layer is the database layer in which the raw sensor data is stored. The raw data is semantically processed in the next layer, the ontology layer. In the ontological layer we have developed a general ontology to describe the different concepts involved in the road traffic scenario, such as vehicles, infrastructure elements, sensors, driver behaviors; and also the relationships between them and a set of reasoning rules applied to the ontology. The final layer is the agent layer, in which the different agents perform all the tasks to improve the driving process. For the accomplishment of each task, the agents exchange information among themselves and with the ontology.

We note that the whole architecture mirrors a holonic traffic architecture [39] in the sense that sensors, vehicles, weather components, and agents, are hierarchically structured.

### 3.1. Sensor Network Layer

We distinguish several types of sensors: agricultural sensors, bridge sensors, road sensors, river monitoring sensors, tunnel sensors, crowd flow detectors, and traffic flow sensors. In particular, the traffic flow sensors are structured as a sensor network [40] and composed of a large number of sensor nodes, densely deployed within the vehicles and on the routes. The sensors deployed on the routes are a high performance, low-cost USB ultrasonic proximity sensor (MB8450) [41] designed to detect the side of a vehicle that drives near the sensor. Such sensor is equipped with a simultaneous multi-sensor feature that allows the sensor to operate even in the presence of other ultrasonic sensors. It is possible to integrate many sensors into one system with a minimal effect from the sensor-to-sensor interference.

Overall, such sensor networks monitor a wide variety of ambient conditions that mainly relate to weather conditions as well as vehicle flow. The flow sensors are in fact detectors that are positioned in particular locations of any lane on the map. They allow the detection of a vehicle’s position, its wheelbase and the time of detection. When performing simulations, it is possible to generate the detection data by sampling from the simulated data on a regular temporal basis in particular lane positions.

In addition to the external sensors, we also distinguish the sensors that are located within the cars, and specific to temperature and humidity.

### 3.2. Data Layer

This is the part where data is collected, transformed, and stored in order to be used in the ontology or agent layer. Herein, we assume that our sensors are distributed around a particular geographic area, collecting different types of data. The goal is to have a real-time dashboard of this data and enable an ad-hoc analysis of historical data merged with weather data. The sensor data is gathered from several resources with a specific data format requirement.

The main data source is relative to the traffic and vehicles flow. Each vehicle is periodically detected on the route and its most important features are stored. Such features include the time at which the detection is performed, the coordinates (latitude and longitude), the vehicle’s speed, velocity angle, the destination, and the wheelbase. The flow is generated by adding extra features such as the current lane, its length, and the average vehicle flow. Additionally, flow detectors are arbitrary assigned to the lanes in a way that samples the current traffic by producing a list of the vehicles that are detected and the relative time.

The sensor data will have to be collected in a highly scalable fashion, in order be transformed and analyzed using the cloud platform. Additionally, the weather data will have to be accessible through remote calls. For such, it is possible to use Google Weather services, OpenWeatherMap, or any other proprietary service. The data collection is performed using a data pipeline that relies on the Google App Engine, and the Google Cloud Endpoints to describe and host our API (Application Programming Interface) endpoint [42]. The APIs are provided to the client-developers (Android for instance) and used in the vehicles for navigation as well as for the reporting of internal conditions change. Additionally, a Google Storage bucket is used to store the collected sensor data and will be used later in the tutorial for moving data to Google BigQuery. As for the manipulation of Big Data, we load the sensor data into Google BigQuery for analysis and visualization, after transforming the data and merging it with weather data [42].

Additionally to the flow data, we distinguish the weather data. Herein, the weather conditions are relative to the city where the traffic is being under consideration. A particular entry to the weather database contains the coordinates of the city center, that is, the longitude and latitude. The main parameters of weather are as follows:

- Temperature and minimum/maximum temperatures that could be reached.
- Atmospheric pressure on the sea level.
- Percentage of humidity.
- Atmospheric pressures on the sea and ground levels.
- Wind speed and direction.
- Cloudiness.
- Rain and snow volumes for the last 3 h.
- Time of data calculation.

### 3.3. Ontology Layer

In the ontology layer of the system, an ontology that relates the different road traffic entities has been developed. For the development of the ontology we have use the Japanese traffic regulations. The ontology was implemented in OWL-RDF language [43] using the *Protégé* tool [44]. In this work we used the reasoner Pellet [45], which is implemented in Java; it is freely available and allows checking the consistency of the ontology.

For better understanding, we present the knowledge in the traffic ontology as three groups of interrelated concepts. The first group contains the elements related to the vehicles, the second group contains the elements related to road infrastructure and the third group includes the concepts related to the sensor subdomain. We explain the details of the three groups below.

#### 3.3.1. Concepts Related to Vehicles

**Figure 2:**
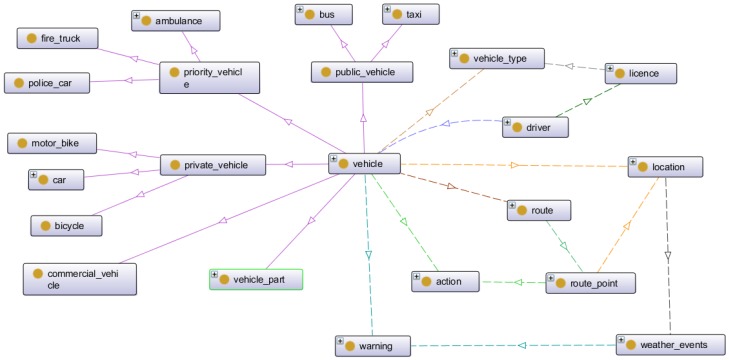
Concepts related to vehicles.

The first group is related to vehicles. The concepts of this group are shown in Figure 2. The figure shows the taxonomy of vehicles, which can be classified into: commercial vehicles, public vehicles (bus and taxi), private vehicles (car, bicycle and motorbike) and priority vehicles (ambulances, fire trucks and police cars). Different relationships between vehicles and other entities are also defined in this group. Some of these entities are: location, which represent the exact latitude and longitude of a vehicle, route point or infrastructure item; information about drivers and the vehicle’s types which they can drive by license. The principal relations defined in this group are: *has_vehicle_type* (vehicle, vehicle_type), *has_driver* (vehicle, driver), *has_licence* (driver, licence), *can_drive* (licence, vehicle_type), *has_route* (vehicle, route), *has_route_point* (route, route_point), *has_location* (vehicle, location), *has_location* (route_point, location), *has_location* (weather_events, location), *has_action* (vehicle, action), *has_ation* (route_point, action), *has_warning* (vehicle, warning), *has_warning* (weather_events, warning).

One of the most important issues in this group is that each vehicle has associated a set of actions, which may vary depending on the route and traffic signals found, and a set of warnings depending on the weather situation in the area.

#### 3.3.2. Concepts Related to Infrastructure

**Figure 3:**
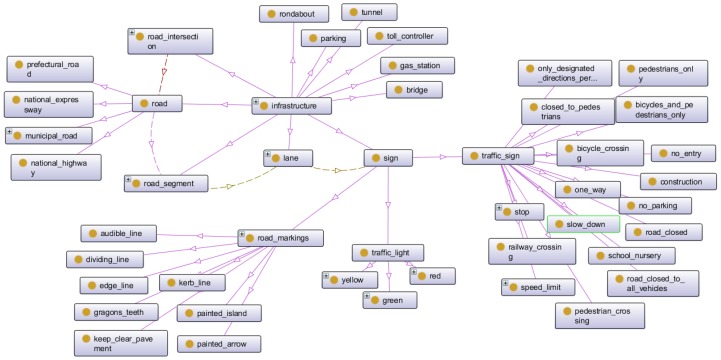
Concepts related to infrastructure.

The second group organizes the elements related to road infrastructure, as shown in Figure 3. In this group the most important concept represents the roads, which, in Japan, are classified as local roads, prefectural roads, national highways and national expressways.

For a better management of traffic situations we divided the roads into segments, connected through intersections. Each segment contains lanes, and different signs such as stop signs or speed control, traffic lights or road markings are per lane. Thus, the principal relations in this group are: *IsOnLane* (sign, lane), *IsOnSegment* (lane, road_segment), and *IsOnRoad* (road_segment, road). Each sign has an action associated following the Japanese traffic regulations.

#### 3.3.3. Concepts Related to Sensors

**Figure 4:**
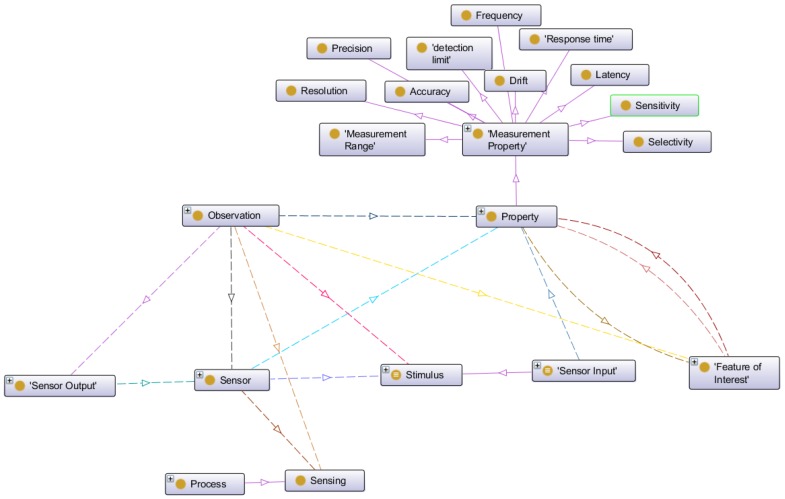
Skeleton of SSN ontology.

The third group deals with the concepts related to the different types of sensors used in the intelligent transportation system scenario. To manage the knowledge related to the sensor domain we have used the Semantic Sensor Network ontology (SSN) which can model sensor devices, systems, processes, observations and knowledge of the environment. The SSN ontology was developed by the Semantic Sensor Networks Incubator Group [46] belonging to the World Wide Web Consortium (W3C). Figure 4 shows the principal concepts in the skeleton of the SSN ontology.

In this ontology the concept *stimulus* represents detectable changes in the environment. In fact, a stimulus is produced by a sensor.

*Sensors* transform an incoming stimulus into another. The observable properties of the sensors include their survival range or accuracy of measurement under defined external conditions. Many sensors need to keep track of time and location to produce meaningful results and, hence, are combined with further sensors to sensor systems such as weather stations [45]. In the case of ITS, the sensors can be located into the vehicles as in different infrastructure elements, for example as part of bridges, roads, signs, etc. The principal sensors we use in this work are related to environmental measures and to flow detection. As environmental sensor we have snow and fog detectors, temperature and humidity sensors. Regarding flow detection we use crowd, pedestrians and car sensors.

*Observations* act as the connection between incoming stimulus, the sensor, and the sensor output. *Properties* are qualities that can be observed by a certain type of sensors. *Features of Interest* are entities in the real world that are the target of sensing. The features have to be fixed by the sensing procedure. *Sensing* is a description of how a sensor works, i.e., how a certain type of stimuli is transformed to a digital representation, perhaps a description of the scientific method behind the sensor.

Sensing methods can also be used to describe how observations where made: e.g., how a sensor was positioned and used. *Sensor Output* represents the result of the observation. The results can act as stimuli or input for other sensors.

### 3.4. Mapping the Sensor Data to Semantic Data

Integration and sharing the sensor data become a big challenging problem in the application of sensor networks. In order to make full use of the sensor data, we need to convert that sensor data into semantic data, which can be understood by computers.

In this work we used a similar procedure as described in [47]. They proposed a mapping file schema, which includes the labeling information of the data sources and the sensor data. The schema falls into two parts: *SourceMapping*, which is used to describe the source information and indicate the key columns (e.g., location name, sensor id, observation value, etc.) to map with Semantic Sensor Networks (SSN) ontology; and *SensorAnnotation*, which is used to annotate every sensor, with concrete sensor type, unit and location.

The first step is to create a mapping file manually, based on the mapping file schema. As the final step, they proposed an algorithm that takes the mapping file as input, and use the predefined correspondences between the mapping schema and SSN ontology to transform sensor data to RDF. Examples of correspondences between the elements in the mapping schema and the classes in SSN ontology are listed in Table 1.

**Figure 5:**
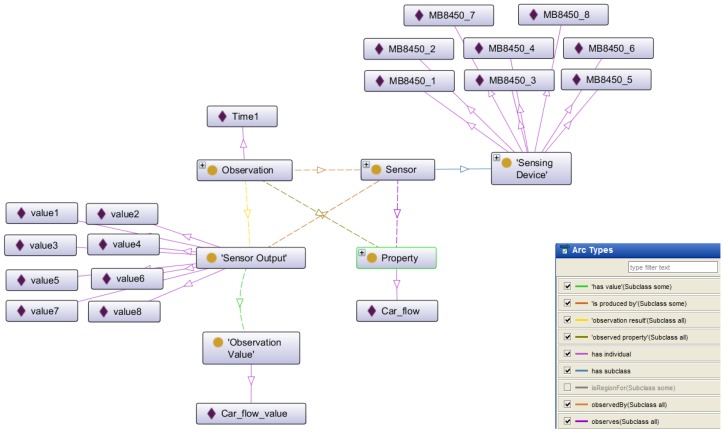
Instance of SSN ontology.

After the last step, the output RDF file represents the instance of the SSN ontology according to the sensor data. Figure 5 shows some of the most important elements (concepts, individuals and relations) of the instances from SSN ontology, generated in the traffic light adjustment scenario. The right corner of the figure shows the types of the relations (arcs) identified in this part of the ontology with different colors. As we can see in this view, the sensing devices observes the *Car_flow* property, producing sensor outputs, with different values.

### 3.5. Agent Layer

At the top layer is the multi-agent system. Here the different agents perform their tasks using the information stored in the ontology through different queries. The multi-agent platform was developed using de Java Agent Development Framework (JADE) [48], thus, the FIPA ACL protocol for agent interactions was used. SPARQL [49] has been used as ontology query language.

In this work we focused in two principal scenarios: the control of air conditioner settings and the traffic light time and intensity adjustment taking into account the traffic flow and the weather conditions. Both scenarios are explained in more detail below. The agents that we have defined in the system for these scenarios are:

- Driver Personal Agent: This agent is responsible of performing the driver personal tasks, taking into account the driver preferences and behavior.
- Environment Agent: This agent is responsible of providing the information related to the environment, measured by the vehicle sensors, for example the temperature and the humidity values.
- Air Conditioner Agent: The principal task of this agent is managing the air conditioner settings, taking into account the values of temperature and humidity measured inside and outside the vehicle and the driver’s preferences.
- Car Agent: This agent was defined to perform in an automated way the different actions related to the car movements following the specific route, the traffic signs and traffic regulations.
- Road Agent: This agent is responsible of performing the tasks related to the road, for example, determine if the road is congested taking into account the information of the traffic flow collected by sensors.
- Traffic Light Agent: This agent perform the tasks related with the traffic light settings, such as: adjusting the duration of the traffic light taking into account the traffic flow and the weather conditions.
- Weather Agent: This agent performs the tasks related to the weather conditions, for example provide the weather information to the rest of the agents of the system and making predictions related to the weather.

#### 3.5.1. Air Conditioning Scenario

**Figure 6:**
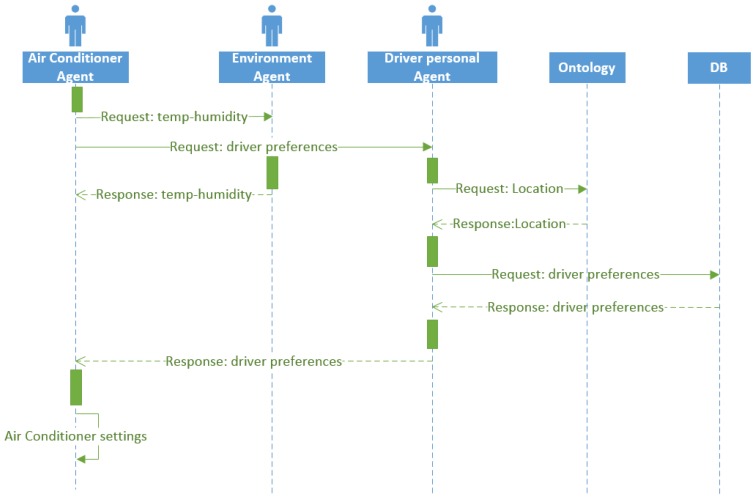
Sequence diagram of the air-conditioner setting task.

This is a simple example scenario that consists in regulating the air conditioning of the car for better comfort while driving. Three agents are involved in this task, the air-conditioner agent, the environment agent and the driver personal agent. To perform this task, the temperature and humidity sensors located in the vehicles are needed. Figure 6 shows the sequence diagram of the air conditioner setting task.

The air conditioning regulation task consists in compare the temperature and humidity measured inside the car with the temperature and humidity outside. With these values and the established values of the driver’s preferences (managed for the driver personal agent), the air-conditioner agent is able to make the decision of adjusting the temperature and humidity of the car. The accomplishment of this task facilitates a comfortable driving ambient.

First, in this task, the air conditioner agent sends two request messages regarding temperature and humidity, one asking the environment agent for the temperature and humidity values and the other asking the driver personal agent for the driver preferences. A person may have different preferences in terms of temperature depending on where he/she is located.

Therefore, once the personal agent receives the request, he consults with the ontology to know the location of the person. Then, knowing the location, the agent queries the database for the user preferences for that particular location and sends that values to the air conditioner agent. Finally, knowing the driver preferences and the environment temperature and humidity values, the air conditioner sets the right combination of parameter values to ensure user comfort.

#### 3.5.2. Traffic Light Adjustment Scenario

**Figure 7:**
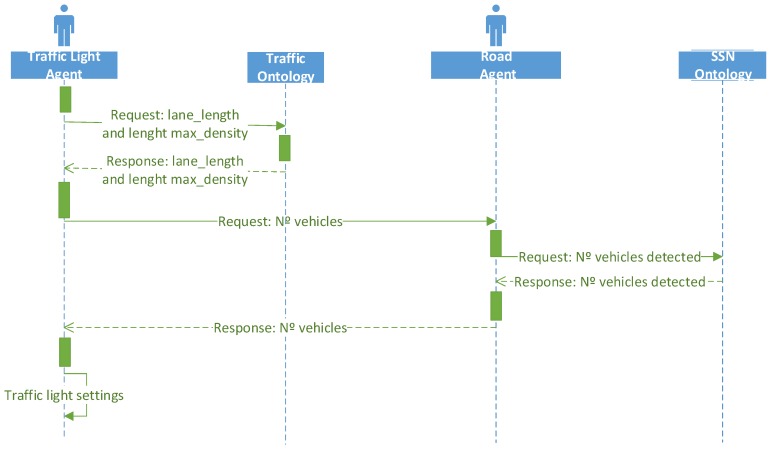
Sequence diagram of the traffic light adjustment task.

The second use case is related to the traffic light adjustment, taking into account the traffic flow and the weather conditions. Figure 7 shows the sequence diagram of the traffic light adjustment scenario. To accomplish the traffic flow detection task, first the agent queries to the traffic ontology to obtain the information about the segment and lane where the traffic light is located. The ontology contains detailed information on each segment and lane, such as its location (latitude and longitude) and the maximum density of the lane. From the coordinates of the beginning and end of each lane segment, using the SWRL rules, the reasoner can infer the length of the lane.

As we explained before, the raw data collected by the sensors is translated into semantic knowledge and stored in an instance of SSN ontology to be processed by the agents. In this case, the road agent makes queries to the SSN ontology to retrieve the information about the number of vehicles detected by the current road sensor.

Taking into account the length of the lane and the number of vehicles detected by the road sensor, the traffic light agent can compute the real density of the lane. The density is defined as the number of vehicles per unit length of the roadway at a specific time [50]. The equation of the density is the following:

$$density=\frac{N}{L}\;,$$

where *N* is the number of vehicles detected by the road sensor in the specific lane, and L is the length of the lane. Once the lane density is computed, the value is compared to the maximum density. If the actual density is greater or equal than the maximum density, it is assumed that lane is congested. Table 2 shows the different rules applied to adjust the traffic lights duration time, taking into account the color of the light and the level of congestion of the road.

As it is shown in the table, if the traffic light is red, and the road segment is congested, then the duration time of the light is decreased; however, if the traffic light is red and the road segment is not congested, then the duration time of the light is maintained in the default value. If the traffic light is green and the road segment is congested, then the duration time of the light is increased, while if it is not congested, the duration time is maintained.

#### 3.5.3. Adaptive Route Management Scenario

In this section we will outline a third use case, in this case for route management, based on our previous work on the use of bio-inspired artificial intelligence for traffic congestion control [51]. The sequence diagram for this scenario would be very similar to the one in Figure 7, although in this case the interaction would happen between a GPS Navigation Agent and the Road Agent. In particular, our traffic congestion control strategies rely on road-provided stigmergy information to be used by GPS navigators to compute minimal-cost routes. There would be two kind of interactions in order to implement the different strategies proposed in [51]:

- Request Road Segment information: The GPS Navigator Agent would request the Road Agent to provide the corresponding information for a particular kind of stigmergy (*short-term*, *long-term* or *anticipatory* as defined in [51]). For both short-term and long-term stigmergies, the information would be in turn requested to the SSN ontology, since it would be provided by sensors in road lanes. The information for anticipatory stigmergies would be provided directly by the GPS Navigator Agents via the interaction below.
- Provide Estimated Position: The GPS Navigator Agent would provide the Road Agent with its estimated position for the pre-established time interval (five minutes in [51]).
- These interactions would allow the GPS Navigator agents to have up-to-date traffic information to be used as weights for route computation. Also, as suggested in one of our recent works [52], negotiation and graph analytics techniques could be used to provide different information to different group of vehicles, effectively managing traffic flows in a semi-decentralized manner.

## 4. Experiments

**Figure 8:**
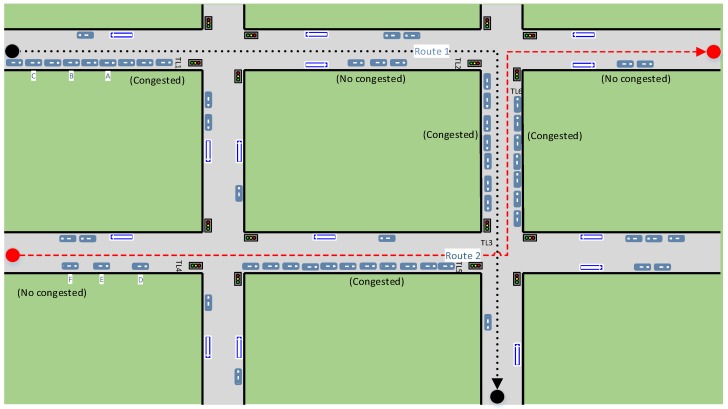
Traffic light duration time scenario.

We have performed several experiments by simulating different traffic scenarios. The following is an example of a simple experiment to probe the automatic synchronization of the traffic light duration time. Figure 8 shows a simple traffic scenario composed of four intersections and four traffic lights on each. The system, taking into account the location and speed of the vehicle, and also the length of each lane segment and the traffic light duration time, can estimate the arrival time of each vehicle to destination, according to the route.

The description of the whole traffic scenario (e.g., roads, lanes, traffic signs and infrastructure elements) is provided by the traffic ontology, while the information concerning the sensors is provided by the SSN ontology. The different agents obtain the knowledge from the ontologies through SPARQL queries.

Two routes were defined in this example, the first one is shown with black dotted lines and the second one is shown with red dashed lines. As we can see in the figure, vehicles A, B and C will follow route 1 and vehicles D, E and F will follow route 2.

For this experiment we have assumed that all lane segments have 200 m length and all vehicles have a constant speed of 60 km/h. Three traffic lights are involved in each route (TL1, TL2, TL3 are the traffic lights in route 1, while TL4, TL5, TL6 are those involved in route 2). The initial configuration of the traffic lights state and the congestion state of the lane segments where they are located is shown in Table 3.

As we can see in the table, at the beginning the first segment of route 1 (S11) is congested and the traffic light located in that segment (TL1) is in red; the second segment of route 1 (S12) is not congested and the corresponding traffic light (TL2) is in red too; the third segment of route 1 (S13) is congested and the traffic light located in that segment (TL3) is in green.

Regarding route 2, the first segment (S_21_) is not congested and its traffic light (TL4) is in red; the second segment (S_22_) is congested and the traffic light located in that segment (TL5) is in red and finally the last segment (S_23_) is also congested but the corresponding traffic light (TL6) is in green.

Table 4 shows the arrival time for the 6 vehicles with the intelligent traffic light time adjustment, and also their arrival time with the default fixed traffic light duration time. The default duration time of the traffic light states was fixed in 15 s. As we can see in the table the total time from the origin to destination for each car is considerably less in the case of intelligent traffic light adjustment.

Vehicles A, B and C will follow route 1. At the beginning, the car A is located at a distance of 100 m of the traffic light, the car B is at 125 m and the car C is at 150 m of the traffic light. The first segment (S11) is congested, so the duration of the red traffic light (TL1) is decreased to 10 s. Therefore, the total time it takes for each car in the first segment S11 is computed as the time it takes to get to the traffic light, plus the duration time of the red light (10 s).

The second segment is not congested, which maintains the default traffic light duration time (15 s). As the three cars have to travel the same distance (200 m), when car A and car B reach the segment, they find the traffic light (TL2) in green. However, when car C, reach the segment, the traffic light has been changed to red, so the time lapse for car C through this segment is higher.

Something similar occurs in the third segment. Vehicles A and B find the TL3 traffic light in green state (the duration of this state is 20 s because the segment is congested), while vehicle C find the traffic light in red color in the same segment. The same situation happens with the vehicles D, E and F, but following the route 2. As we can see in the table, the vehicles took considerably less time on each segment in the case in which the intelligent adjustment of the traffic light duration time was applied. In case of congestion, the vehicles have to wait for shorter periods of time at red lights, while they can move for longer periods of time with green lights. This adjustment gives higher priority to congested traffic segments over the rest of the traffic to improve the traffic flow and to avoid traffic jams.

In this work, 150 experiments were conducted, with 300 vehicles for which different traffic scenarios were defined. These scenarios were composed of 50 routes within a radius of 10 km, with 10 intersections regulated by traffic lights (40 traffic lights). For each experiment, the chosen initial configuration for traffic lights, and the position and route of the vehicles was random. Table 5 shows the results obtained in the experiments. The table also shows the percentage of vehicles that gained time using the automatic synchronization of traffic lights; the average of time gained by vehicle; and the percentage of vehicles that did not gain time. As we can see in the table, 78% of vehicles experienced a gain of time, and the average value of gained time was approximately 134.52 s. No vehicle experienced a loss of time in the experiments, while vehicles that did not gain time (22%) were those who moved along uncongested routes.

## 5. Conclusions

This paper presents an ontology-based architecture to improve the driving environment through a traffic sensor network. The system performs different tasks in an automatic way to increase the driver safety and comfort using sensor information. The proposed system is a four layer architecture. At the bottom is the sensor network, deployed along the road infrastructure. In the second layer is the database in which the raw sensing data will be stored. The raw data is related semantically in the ontology layer. In this layer we have developed a general ontology to describe the different concepts involved in the road traffic scenario, and also the relations between them and a set of rules for reasoning with the ontology. At the top is the agent layer, in which the different agents perform all the tasks to improve the driving process. Examples of these tasks are the air conditioner parameters’ setting and the adjustment of the traffic light duration time, taking into account the traffic flow and the weather conditions. This system allows us to give higher priority to road segments that are congested over others.

Ontologies can describe the current traffic situation, including the location of vehicles, infrastructure elements and sensors; and it allows us to analyze semantically the data collected by the sensors. One of the advantages of our proposal is that agents can know the overall traffic situation at all times by querying the ontology, instead of having only a partial view (and therefore imprecise). This allows greater decision-making capability based on the real circumstances, and that agents can work together using the same knowledge base.

Several experiments have been performed to simulate actual road traffic situations. It has been shown that the automatic adjustment of the duration of the traffic lights contributes to optimizing the traffic flow, which allows drivers to gain time along the route. To carry out this process, the system takes into account only the level of congestion and the weather conditions of the corresponding road segment.

Although the performed experiments have yielded satisfactory results, there is still plenty of work to be done in this area. As discussed when describing the third traffic scenario, there are many other factors involved in this process, which have not been considered in the experiments, such as the congestion level of other road segments along the route. We want to implement this third scenario in our simulator and carry on further experiments, integrating this line of work with our previous and current work on negotiation techniques in traffic scenarios.

## Figures and Tables

**Table 1:** Correspondences between the elements in the mapping schema and the classes in SSN (from [47]).

Mapping Schema Element	SSN Class	Description
location name	ssn:Deployment	location of the sensors
sensor id	ssn:Sensor	number of the sensors
type	ssn:Property	type of the sensors
observation value	ssn:SensorOutput	output of the sensors
unit	ssn:UnitOfMeasure	unit of the data
text of observation value	ssn:ObservationValue	values of the observation
observation time	ssn:Observation	time of the sensor data

**Table 2:** Rules applied to adjust the traffic light duration time.

	Congested	Not Congested
Red	Decrease	No change
Green	Increase	No change

**Table 3:** Initial configuration of the traffic lights and segment states for the simulation.

S_11_-TL1	S_12_-TL2	S_13_-TL3	S_21_-TL4	S_22_-TL5	S_23_-TL6
Congested	Not congested	Congested	Not congested	Congested	Congested
Red	Red	Green	Red	Red	Green

**Table 4:** Time the vehicles take to move through the different road segments.

	Time with Traffic Light Duration Adjustment					Time with Default Traffic Light Duration				
	S_11_	S_12_	S_13_	S_14_	Sum	S_21_	S_22_	S_23_	S_24_	Sum
A	6 + 10 = 16	12	12	12	52	6 + 15 = 21	12 + 15 = 27	12 + 15 = 27	12	75
B	7.5 + 10 = 17.5	12	12	12	53.5	7.5 + 15 = 22.5	12 + 15 = 27	12 + 15 = 27	12	76.5
C	9 + 10 = 19	12 + 15 = 27	12 + 10 = 22	12	80	9 + 15 = 24	12 + 15 = 27	12 + 15 = 27	12	88
D	4.2 + 15 = 19.2	12 + 10 = 22	12 + 10 = 22	12	75.2	4.2 + 15 = 19.2	12 + 15 = 27	12 + 15 = 27	12	85.2
E	6 + 15 = 21	12 + 10 = 22	12	12	67	6 + 15 = 21	12 + 15 = 27	12	12	72
F	9 + 15 = 24	12 + 10 = 22	12	12	70	9 + 15 = 24	12 + 15 = 27	12	12	75

**Table 5:** Overall results of the experiments in simulation, showing the percentages (%) of vehicles experimenting and not experimenting time gains, along with the average gain in time.

Number of Experiments	Number of Vehicles	% Gain Time	Average Gained Time	% Not Gain Time
150	300	78%	134.52 s	22%
